# Therapeutic hybrid intelligence with neural and knowledge-based expert reasoning for SRS: an AI model for breast cancer brain metastases

**DOI:** 10.3389/fonc.2026.1884891

**Published:** 2026-09-11

**Authors:** Jheremy S. Reyes, Ajay Niranjan, Constantinos G. Hadjipanayis

**Affiliations:** 1 Center for Image-Guided Neurosurgery, Department of Neurological Surgery, University of Pittsburgh Medical Center, Pittsburgh, PA, United States; 2 Computational Neurosurgery Research Group (CIGNS-CRG), Center for Image-Guided Neurosurgery, Department of Neurological Surgery, University of Pittsburgh Medical Center, Pittsburgh, PA, United States

**Keywords:** artificial intelligence, breast brain metastases, dose optimization, local failure, stereotactic radiosurgery

## Abstract

**Background:**

Prescription dose selection for breast cancer brain metastases treated with stereotactic radiosurgery remains largely guided by tumor size, anatomical constraints, and institutional practice rather than individualized tumor-specific estimates of local failure. We developed THINKERS-Breast, a mixture-of-experts artificial intelligence framework for personalized dose evaluation after Gamma Knife radiosurgery.

**Methods:**

We performed a retrospective single-center tumor-level study of breast cancer brain metastases treated with Gamma Knife radiosurgery. Variables available before or at treatment were used to train a mixture-of-experts neural network with discrete-time survival modeling. Margin dose was incorporated as a queryable input to enable repeated candidate dose evaluation. Internal validation used grouped 5-fold cross-validation and a grouped holdout test split by patient. Performance was assessed using area under the receiver operating characteristic curve (AUC) for 12-month local failure, mean absolute error (MAE) for expected time to local failure, Brier score, and calibration metrics.

**Results:**

The cohort included 3,098 tumors from 504 patients. In grouped cross-validation, THINKERS-Breast achieved raw mean AUC >0.807 and calibrated mean AUC of 0.864 for 12-month local failure. Raw and calibrated Brier scores were <0.14 and <0.16, respectively, with calibration intercepts ranging from -0.31 to +0.27. In the grouped holdout set, AUC was 0.781 (95% CI, 0.704–0.857), and MAE for expected time to local failure was 1.55 months (95% CI, 0.78–3.42).

**Conclusions:**

THINKERS-Breast provides an internally validated framework for tumor-specific local failure prediction and dose-policy evaluation after Gamma Knife radiosurgery for breast cancer brain metastases. External validation is required before clinical deployment.

## Introduction

Breast cancer brain metastases are a frequent and clinically important indication for stereotactic radiosurgery (SRS) in contemporary neuro-oncologic practice (1–3). Brain metastases occur in approximately 20–40% of patients with breast cancer during the course of disease, with particularly high central nervous system involvement among HER2-positive and triple-negative subtypes (4, 5). In metastatic HER2-positive or triple-negative breast cancer, nearly one-third of patients may develop brain metastases during their lifetime, and some contemporary series report cumulative incidences approaching 30–50% in high-risk biological subgroups (6, 7). As systemic therapies have improved extracranial disease control and prolonged survival, durable intracranial tumor control has become increasingly important.

Gamma Knife radiosurgery (GKRS) is an established treatment option for breast cancer brain metastases and can provide meaningful local control while avoiding or delaying the neurocognitive risks associated with whole-brain radiotherapy (4–7). However, prescription dose selection remains largely guided by institutional practice patterns, tumor size, anatomical location, proximity to critical structures, and physician experience rather than individualized estimates of tumor-specific behavior after treatment (7, 8). This is an important limitation because breast cancer brain metastases are biologically and clinically heterogeneous, with outcomes influenced by tumor volume, intracranial disease burden, systemic therapy exposure, breast cancer subtype, anatomical context, baseline functional status, and radiosurgical dose characteristics. In routine practice, these factors are rarely integrated into a formal framework for personalized dose evaluation.

**Figure 1:**
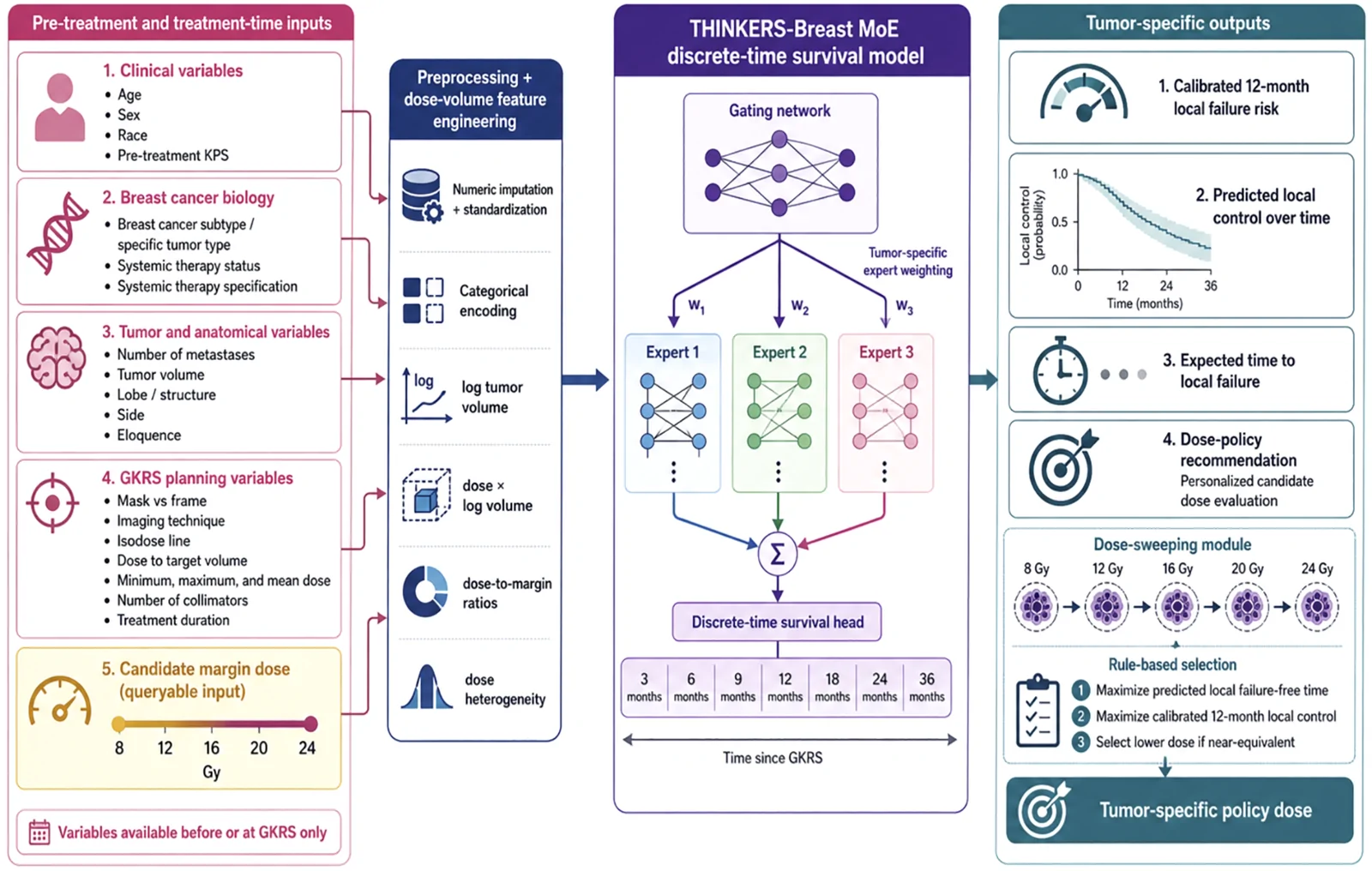
THINKERS-Breast architecture. Schematic overview of the THINKERS-Breast framework. Pre-treatment clinical, breast cancer subtype, systemic therapy, tumor, imaging, and radiosurgical variables, together with candidate margin dose, are processed through a mixture-of-experts discrete-time survival model. The framework generates calibrated 12-month local failure risk, predicted local control over time, expected time to local failure, and tumor-specific dose-policy recommendations.

Artificial intelligence has increasingly been applied to outcome prediction after stereotactic radiosurgery, including models based on clinical variables, radiomics, deep learning, and combinations of imaging and dosimetric features (9–23). Prior studies have demonstrated the feasibility of predicting local control, local failure, or treatment response after SRS/SRT for brain metastases (19–23). However, most of these approaches have focused on classification of treatment response or local control at a predefined endpoint and generally do not evaluate how the predicted outcome for an individual tumor changes across alternative candidate prescription doses. In addition, many existing models are not designed to provide a complete time-dependent local failure profile within a histology-specific framework. To address these limitations, we developed Therapeutic Hybrid Intelligence with Neural and Knowledge-based Expert Reasoning for SRS in Breast Cancer Brain Metastases (THINKERS-Breast), a breast cancer-specific mixture-of-experts framework with discrete-time survival modeling in which candidate margin dose is incorporated as a queryable input. The framework generates dose-specific estimates of local control, calibrated 12-month local failure risk, and expected time to local failure, enabling lesion-level comparative dose evaluation before treatment (**Figure 1**).

## Methods

### Study design and data source

We conducted a retrospective single-center cohort study at the Center for Image-Guided Neurosurgery, University of Pittsburgh Medical Center (UPMC), including consecutive patients with breast cancer brain metastases treated with GKRS between 2014 and 2024. Clinical, demographic, tumor, systemic therapy, imaging, and treatment-related variables were extracted from institutional records and radiosurgical planning data and compiled into a structured analytic dataset. Each row represented an individual treated brain metastasis and included baseline clinical features, tumor characteristics, radiosurgical parameters, systemic therapy information, and follow-up outcome data. Patients were included if they had a confirmed diagnosis of breast cancer, one or more brain metastases treated with GKRS between 2014 and 2024, available radiosurgical treatment-planning data, documented tumor-level local control status, and available follow-up time after treatment. Tumors were excluded if they lacked essential dosimetric variables, tumor volume, treatment date, local control outcome, or follow-up duration. Patients treated for non-breast primary tumors, tumors treated with fractionated or staged SRS, and observations containing post-treatment variables only without baseline treatment-planning information were excluded from the final analytic cohort.

### Patient, tumor, and treatment characteristics

Baseline variables were restricted to information available at or before the time of GKRS to preserve clinical deployability and avoid post-treatment leakage. The final feature set included age at treatment, race, sex, breast cancer subtype or specific tumor type, pre-treatment Karnofsky Performance Status (KPS), number of metastases, metastasis location, lobe or structure, eloquence, tumor volume, systemic therapy status, systemic therapy specification, mask versus frame immobilization, imaging techniques, isodose line, margin dose, dose to target volume, minimum dose, maximum dose, mean dose, number of collimators, and treatment duration. Additional derived variables were constructed to represent clinically relevant dose-volume interactions, including logarithmic tumor volume, tumor volume category, dose-volume interaction terms, dose-to-volume ratios, dose heterogeneity, and minimum, maximum, and mean dose-to-margin dose ratios. Tumors were classified as involving or adjacent to eloquent or critical anatomy when they were located in or near regions where radiation-related injury could plausibly result in clinically meaningful neurological deficits. These regions included the primary motor cortex, primary sensory cortex, language cortex, basal ganglia, internal capsule, thalamus, brainstem, optic apparatus or visual pathways, and deep cerebellar structures or cerebellar peduncles when clinically relevant. Eloquent status was assigned based on treatment-planning review and documented anatomical location at the time of GKRS.

### Additional clinical and prior treatment variables

Additional clinical and prior treatment variables were extracted when available before or at the time of GKRS. These included prior whole-brain radiotherapy (WBRT), prior stereotactic radiosurgery (SRS), prior neurosurgery, prior targeted therapy, breast cancer recurrence, recurrent brain metastases, symptomatic status at GKRS, prior intracranial treatment failure, and retreatment history. These variables were included as candidate model inputs when available before or at treatment. Categorical variables were encoded using the same preprocessing strategy as the remaining clinical variables, with missing or unavailable values handled during preprocessing to avoid excluding otherwise eligible tumors.

### Treatment protocol

GKRS was performed using Leksell Gamma Knife Icon and Esprit systems according to institutional practice during the study period. Treatment planning was performed using Leksell GammaPlan based primarily on stereotactic contrast-enhanced MRI, with CT used when clinically necessary. The prescription margin dose, isodose line, target coverage, number of collimators, and treatment duration were determined by the treating radiosurgical team according to tumor volume, geometry, anatomical location, proximity to eloquent or critical structures, and prior treatment history. All tumors in the analytic cohort were treated with single-session GKRS. Fractionated or staged SRS treatments were not included in the final analytic cohort. Treatment planning was based on the contrast-enhancing tumor volume, which was contoured as the gross tumor volume (GTV) on stereotactic contrast-enhanced MRI. No additional planning target volume (PTV) margin was routinely added, consistent with standard Gamma Knife radiosurgery practice.

### Outcome definition and follow-up

The primary endpoint was time to local failure after GKRS, modeled under right censoring. Local failure events were defined by documented loss of local control on follow-up imaging. Tumors without documented local failure were treated as censored or locally controlled observations. Follow-up was calculated from the date of GKRS to the date of last available imaging follow-up, documented local failure, or censoring. The last available follow-up date for outcome assessment was 12/31/2024. Follow-up duration was summarized using median and interquartile range, as well as mean, standard deviation, and range. The final framework was designed not only to estimate local failure risk over time, but also to support comparative evaluation of candidate margin doses for individualized dose assessment.

### Predictive model development and validation

Model development used a unified preprocessing pipeline to ensure consistent handling of continuous and categorical variables. Continuous variables were converted to numeric format when appropriate, imputed using the median, and standardized. Categorical variables were encoded as strings, missing categorical entries were treated as a separate category, and one-hot encoding was applied with support for infrequent and previously unseen levels. Post-treatment variables, including post-treatment KPS, complications, cerebral edema, neurologic deficits, death status, new metastases after treatment, and other follow-up outcomes, were excluded to prevent leakage (**Supplementary Tables 1**, **2**).

We developed THINKERS-Breast as a deep learning framework based on a MoE neural network with discrete-time survival modeling. The architecture included three expert subnetworks, each implemented as a fully connected feedforward neural network with rectified linear unit activations, batch normalization, and dropout regularization, together with a gating network that assigned tumor-specific weights to the expert outputs. The final network generated interval-specific hazard estimates for local failure across predefined follow-up intervals of 3, 6, 9, 12, 18, 24, and 36 months. These hazard estimates were used to reconstruct local failure-free survival curves and derive secondary outputs, including predicted 12-month local failure probability and expected time to local failure. Margin dose was incorporated as an explicit input variable to enable repeated evaluation across candidate dose levels for personalized dose assessment.

To improve model robustness, the final implementation incorporated clinically plausible feature engineering, weighted loss handling for event imbalance, dropout regularization, early stopping, and an ensemble strategy using multiple MoE models trained with different random seeds. A probability calibration step was applied to improve the clinical interpretability of predicted 12-month local failure probabilities.

### Derived dose-volume feature engineering

Additional derived variables were constructed to capture clinically relevant relationships between tumor size, prescription dose, and delivered dose distribution. These variables were generated only from information available at the time of GKRS planning. Tumor volume was log-transformed to reduce right-skewness and improve modeling of nonlinear size effects. Dose-volume interaction terms were created because the clinical effect of a given margin dose may differ according to tumor size. Dose-to-volume and dose-to-margin ratios were generated to represent relative dose intensity, target coverage, and intratumoral dose heterogeneity beyond the prescription margin dose alone. These engineered features were included to support the model’s ability to learn nonlinear radiosurgical dose-response patterns while preserving clinical interpretability. Full definitions and rationales are provided in **Supplementary Table 3**.

### Modeling strategy and rationale

The mixture-of-experts neural network architecture was selected because the study objective required more than binary risk classification (17). The framework was designed to estimate time-dependent local failure risk, reconstruct tumor-specific local control profiles, and repeatedly evaluate the same tumor across candidate margin doses (17). This required a flexible nonlinear model capable of representing interactions among tumor volume, anatomical location, systemic therapy context, breast cancer subtype, and radiosurgical dose variables. Conventional approaches such as logistic regression, Cox regression, random forest, support vector machines, and gradient boosting were considered as alternative modeling strategies. However, these methods are less naturally suited to simultaneously modeling discrete-time hazards, censored observations, heterogeneous dose-response patterns, and repeated counterfactual-style candidate dose queries within a single unified framework. The MoE structure was therefore selected to allow multiple expert subnetworks to learn partially distinct feature-dose-outcome relationships, while a gating network adaptively weighted expert contributions for each tumor (17).

### Model architecture and training hyperparameters

The THINKERS-Breast architecture consisted of three expert subnetworks and a gating network. Each expert was implemented as a fully connected feedforward neural network with three hidden layers containing 128, 64, and 32 neurons, respectively. Rectified linear unit activations and batch normalization were applied after each hidden layer, together with dropout regularization using a probability of 0.25. Each expert generated interval-specific local failure hazard estimates across the seven predefined follow-up intervals. The gating network consisted of two fully connected hidden layers containing 64 and 32 neurons, followed by a softmax output layer that generated normalized tumor-specific weights across the three experts. The final prediction was obtained by combining the expert outputs according to these gating weights.

Model parameters were optimized using the Adam optimizer with an initial learning rate of 0.001 and a batch size of 64. Training incorporated a weighted discrete-time survival loss to account for event imbalance and right-censored observations. Early stopping monitored validation loss with a patience of 20 epochs, and the model parameters corresponding to the lowest validation loss were retained. To reduce sensitivity to random initialization, multiple MoE models were trained using different random seeds and their predicted probabilities were averaged to generate the final ensemble output.

### Input feature selection

Input features were selected *a priori* according to clinical availability at the time of GKRS, biological plausibility, radiosurgical relevance, and completeness across the retrospective cohort. Patient-level variables were selected to capture baseline clinical status, breast cancer biology, systemic therapy context, extracranial disease status, and prior treatment history. Tumor-level variables were selected to capture intracranial tumor burden, anatomical context, eloquence, tumor volume, and local treatment constraints. GKRS planning variables were selected to represent prescription dose, delivered target dose, dose heterogeneity, treatment complexity, and immobilization or imaging approach. Variables only available after treatment, including post-treatment complications, edema, neurologic deficits, death status, new metastases after treatment, and follow-up outcomes, were excluded to avoid leakage. This strategy was intended to preserve clinical deployability by restricting the model to information that could be known before or during radiosurgical planning.

### Internal validation and performance metrics

Internal validation consisted of a grouped holdout train-test split at the patient level, followed by grouped 5-fold cross-validation to prevent tumor-level leakage across patients. Model performance was assessed by discrimination for 12-month local failure, quantified using the area under the receiver operating characteristic curve (AUC), and by temporal accuracy for expected time to local failure, quantified using mean absolute error (MAE) in months among tumors with observed local failure. Probabilistic performance was assessed using the Brier score, calibration intercept, and calibration slope. Bootstrap resampling of the holdout test set was used to estimate 95% confidence intervals for AUC, Brier score, and MAE.

Although THINKERS-Breast generated interval-specific local failure estimates at 3, 6, 9, 12, 18, 24, and 36 months, the 12-month endpoint was selected as the primary discrimination time point because it represents a clinically relevant interval for post-SRS local control assessment while retaining sufficient follow-up and event information within the retrospective cohort. Earlier intervals contain fewer observed failures, whereas later intervals are increasingly affected by censoring and reduced numbers at risk. The remaining time points were retained within the discrete-time survival framework to reconstruct longitudinal local control profiles and support time-dependent prediction, but were not used as the principal binary discrimination endpoint.

### Personalized dose recommendation framework

To support individualized dose evaluation, margin dose was treated as a manipulable input variable rather than as a fixed historical label. For each tumor, the final trained model was queried repeatedly across a predefined set of candidate margin doses. For every candidate dose, the framework generated a predicted tumor profile, including estimated local control probabilities at clinically relevant horizons, calibrated 12-month local failure risk, and expected time to local failure. Dose recommendation was performed through a rule-based optimization strategy that prioritized predicted local failure-free time, followed by calibrated 12-month local control, while using lower dose only as a final tie-breaker among near-equivalent candidates. This approach was intended to emulate radiosurgical decision-making by identifying the lowest dose that preserved favorable predicted local control dynamics for a given tumor.

### Feature importance analysis

Permutation feature importance was calculated in the grouped holdout test set to quantify the contribution of each input variable or variable group to model discrimination. For each variable, 100 independent permutation repetitions were performed while all remaining input features were held unchanged. At each repetition, the decrease in AUC for 12-month local failure relative to the unpermuted baseline model was calculated, and the mean decrease in AUC across repetitions was used as the final importance estimate.

For categorical variables represented by multiple one-hot encoded columns, all columns corresponding to the same original clinical variable were permuted jointly to preserve the structure of the parent feature and provide clinically interpretable group-level importance estimates. Larger decreases in AUC indicated greater contribution to model discrimination. Relative importance was normalized to the largest observed mean decrease in AUC, with the most influential variable assigned a value of 100%.

### Statistical analysis and ethics

Descriptive statistics were summarized using standard measures. Continuous variables were described using medians and interquartile ranges, with means, standard deviations, and ranges reported where appropriate. Categorical variables were summarized as counts and percentages. All analyses were performed in Python using fixed random seeds to ensure reproducibility. This retrospective study was conducted under institutional oversight in accordance with UPMC requirements, with waiver of consent where applicable.

### Software and computational environment

All analyses and model development were performed in Python version 3.11. Neural network development and training were implemented using PyTorch version 2.3.1. Data preprocessing, grouped cross-validation, receiver operating characteristic analysis, calibration assessment, and permutation feature importance were performed using scikit-learn version 1.5.1. Data manipulation and numerical operations were performed using pandas version 2.2.2 and NumPy version 1.26.4. Statistical and scientific computing procedures additionally used SciPy version 1.14.0. All analyses were performed using fixed random seeds to improve computational reproducibility.

## Results

### Study cohort

During the study period, 613 patients treated with GKRS for brain metastases were screened for eligibility. After exclusion of 109 patients and 227 tumors because of missing essential treatment-planning data, missing tumor volume, unavailable local control outcome, missing follow-up duration, non-breast primary tumor diagnosis, or fractionated/staged SRS, the final analytic cohort included 504 patients with 3,098 breast cancer brain metastases. The dataset was analyzed at the tumor level, with each row representing an individual treated metastasis. The grouped holdout test set included 619 tumors from 100 patients, while the remaining tumors were used for training and internal validation. The median imaging follow-up duration was 12.4 months (IQR, 5.8–27.6), with a mean follow-up of 19.7 ± 22.3 months and a range of 1.5–118.4 months. The last available follow-up date was December 31, 2024.

The cohort included treatment-time demographic variables, pre-treatment functional status, breast cancer subtype or specific tumor type, systemic therapy information, tumor burden, anatomical location, eloquence, tumor volume, immobilization approach, imaging technique, and radiosurgical dose parameters. Treatment-related features included margin dose, isodose line, dose to target volume, minimum dose, maximum dose, mean dose, number of collimators, and treatment duration (**Table 1**). Derived dose-volume features were also generated to capture clinically relevant interactions between tumor size and radiosurgical dose.

**Table 1:** Baseline clinical, tumor, molecular, and Gamma Knife radiosurgery (GKRS) treatment characteristics of the study cohort. .

Characteristic	Value
Cohort size	3,098 tumors, 504 patients
Patient-level characteristics	
Age at treatment, years	60.0 (52.0–70.0); 60.9 ± 12.9; range 30.0–91.0
Sex	
Female	499 (99%)
Male	5 (1%)
Race	
White	475 (94.1%)
African American	24 (4.9%)
Asian	5 (1.0%)
Pre-treatment KPS	70 (60–80); 68 ± 9; range 40–90
Systemic therapy	
Yes	353 (70.1%)
No	151 (29.9%)
Breast cancer subtype/specific tumor type	
Adenocarcinoma	91.5%
Triple negative	4.9%
Triple positive	2.0%
Ductal	0.7%
Other	0.8%
Number of metastases	
Multiple	98.1%
Single	1.9%
Metastasis location	
Supratentorial	62.2%
Infratentorial	37.8%
Lobe/structure	
Cerebellum	31.9%
Frontal	30.1%
Parietal	12.5%
Temporal	8.5%
Occipital	7.2%
Other	9.8%
Side	
Left	56.5%
Right	43.5%
Eloquence	
Yes	53.5%
No	46.5%
Tumor volume, cm³	0.130 (0.034–0.852); 1.288 ± 3.636; range 0.002–50.159
Extracranial metastatic disease	
Yes	504 (100%)
No	0 (0%)
GKRS treatment characteristics	
Margin dose, Gy	16.0 (16.0–18.0); 16.6 ± 1.9; range 8.0–27.0
Isodose line, %	80.0 (55.0–85.0); 71.9 ± 16.1; range 50–90.0
Number of collimators	22 (14–29); 23 ± 16; range 1–124
Treatment duration, min	112.0 (70.0–147.0); 109.7 ± 46.3; range 9.0–191.0
Imaging technique	
MRI	98.7%
CT	1.3%
Immobilization approach	
Frame-based GKRS	3,050 (98.5%)
Mask-based GKRS	34 (1.1%)
CT-based setup/other	14 (0.5%)
Treatment fractionation	
Single-session GKRS	3,098 (100%)
Fractionated/staged SRS	0 (0%)
Prior treatment and disease-status variables	
Prior WBRT	
Yes	12 (2.4%)
No	492 (97.6%)
Prior SRS	
Yes	118 (23.4%)
No	386 (76.6%)
Prior neurosurgery	
Yes	64 (12.7%)
No	440 (87.3%)
Prior targeted therapy	
Yes	289 (57.3%)
No	215 (42.7%)
Breast cancer recurrence	
Yes	421 (83.5%)
No	83 (16.5%)
Recurrent brain metastasis	
Yes	156 (31.0%)
No	348 (69.0%)
Symptomatic status at GKRS	
Symptomatic	228 (45.2%)
Asymptomatic	276 (54.8%)
Prior treatment failure	
Yes	142 (28.2%)
No	362 (71.8%)
Retreatment history	
Yes	128 (25.4%)
No	376 (74.6%)

Continuous variables are reported as median (interquartile range), with mean ± standard deviation and range. Categorical variables are reported as counts and percentages.

### Model performance in internal validation

The final THINKERS-Breast discrete-time survival MoE model demonstrated strong discrimination for 12-month local failure across grouped patient-level validation. In grouped 5-fold cross-validation, the model achieved a raw mean AUC greater than 0.807 and a calibrated mean AUC of 0.864 for 12-month local failure. Probabilistic performance was favorable, with raw and calibrated Brier scores below 0.14 and 0.16, respectively. Calibration was acceptable, with raw calibration intercepts ranging from -0.31 to +0.27.

### Holdout test performance

**Figure 2:**
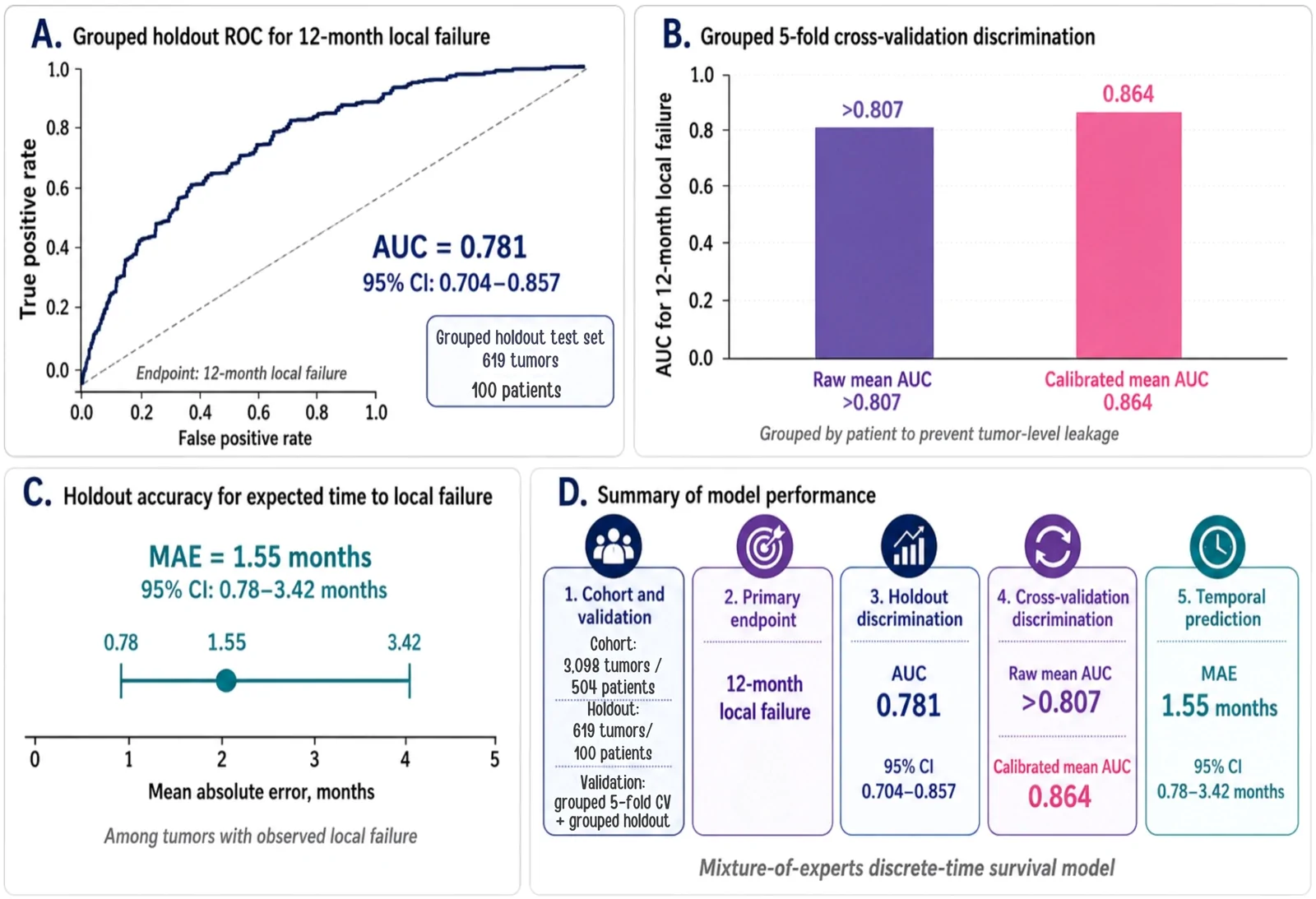
Internal validation and holdout performance. Performance of THINKERS-Breast for 12-month local failure prediction. The model achieved an AUC of 0.781 in the grouped holdout test set, with a bootstrapped 95% CI of 0.704–0.857. Grouped 5-fold cross-validation showed strong discrimination, with raw mean AUC >0.807 and calibrated mean AUC of 0.864. Expected time to local failure was predicted with a mean absolute error of 1.55 months.

On the grouped holdout test set, THINKERS-Breast achieved strong discrimination for 12-month local failure, with an AUC of 0.781 and a bootstrapped 95% confidence interval of 0.704–0.857. Predicted time to local failure showed a mean absolute error of 1.55 months, with a bootstrapped 95% confidence interval of 0.78–3.42 months. These findings indicate that the final discrete-time survival formulation retained meaningful performance in unseen patients and tumors while providing individualized estimates of local failure timing among tumors with observed events (**Figure 2**). In additional holdout performance analyses, THINKERS-Breast achieved a C-index of 0.81 for time-to-local-failure prediction. Using the Youden index threshold for 12-month local failure classification, sensitivity was 80% and specificity was 77%, supporting balanced discrimination between tumors with and without 12-month local failure.

### Probabilistic performance and calibration

**Figure 3:**
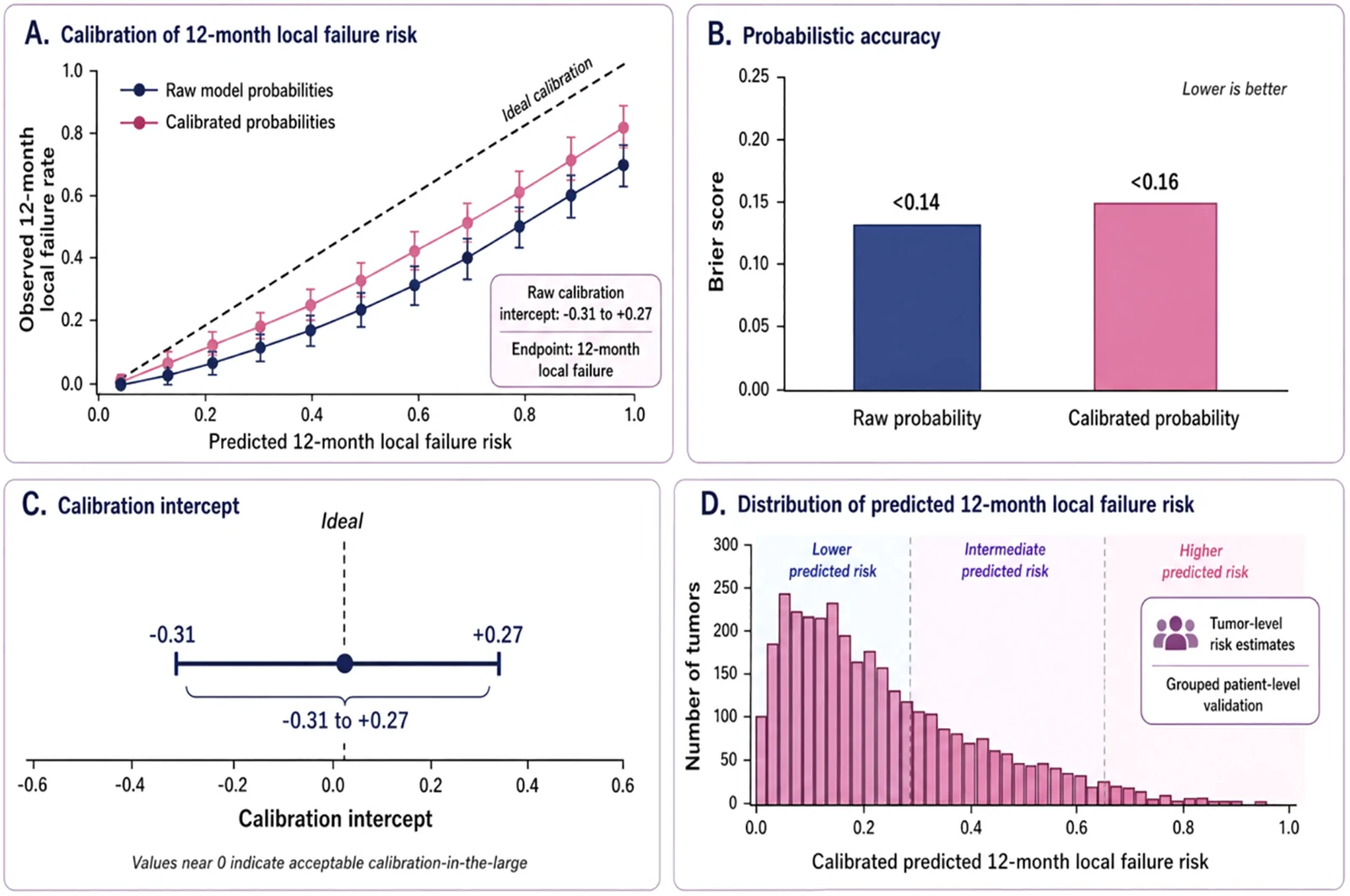
Calibration and probabilistic performance. Probabilistic performance of THINKERS-Breast for 12-month local failure prediction. Calibration analysis demonstrated acceptable calibration-in-the-large, with raw calibration intercepts ranging from -0.31 to +0.27. Raw and calibrated Brier scores were below 0.14 and 0.16, respectively, supporting favorable probabilistic accuracy.

Probabilistic performance was favorable after calibration. The final calibrated Brier score was below 0.16, while the raw Brier score remained below 0.14. Raw calibration intercepts ranged from -0.31 to +0.27, suggesting acceptable agreement between predicted and observed 12-month local failure risk (**Figure 3**).

### Dose recommendation framework

Using the final trained MoE model, tumor-specific dose evaluation was performed by repeatedly evaluating candidate margin doses and reconstructing dose-dependent local failure-free survival profiles. For each tumor, the framework generated calibrated 12-month local failure risk, predicted local control probabilities across clinically relevant time intervals, and expected time to local failure. Dose recommendation was then performed using a rule-based optimization strategy that prioritized predicted local failure-free time, followed by calibrated 12-month local control, with lower dose used only as a final tie-breaker among near-equivalent candidates. Qualitative review of the resulting recommendations showed clinically plausible tumor-level dose variation, supporting the feasibility of THINKERS-Breast as an individualized dose-policy auditing framework for breast cancer brain metastases treated with GKRS.

### Feature importance analysis

Permutation feature importance analysis showed that THINKERS-Breast predictions were driven primarily by radiosurgical dose parameters, tumor volume, and derived dose-volume interaction features. Permutation of margin dose resulted in the largest decrease in AUC, followed by tumor volume and dose-volume interaction features. Dose-distribution variables, including mean dose, dose to target volume, maximum dose, dose heterogeneity, minimum dose, and minimum-dose-to-margin ratio, also contributed substantially to model discrimination. Clinical and biological variables, including number of metastases, anatomical location, eloquence, breast cancer subtype, systemic therapy status, pre-treatment KPS, and age, provided additional predictive information. Variables with limited variability, including immobilization approach, imaging technique, sex, and race, had lower relative importance. The complete permutation feature importance analysis is provided in **Supplementary Table 2**.

### Illustrative case

As an illustrative example of how THINKERS-Breast may be applied in practice, we present a case of a 54-year-old woman with HER2-positive metastatic breast cancer who underwent GKRS for five brain metastases identified on surveillance MRI. The patient had preserved functional status, controlled extracranial disease on systemic therapy, and no prior whole-brain radiotherapy. The tumors differed substantially in volume and anatomical context: a 0.04 cm³ right frontal tumor, a 0.22 cm³ left cerebellar tumor, a 0.63 cm³ right parietal tumor near eloquent cortex, a 1.48 cm³ left temporal tumor, and a 2.85 cm³ deep thalamic tumor.

**Figure 4:**
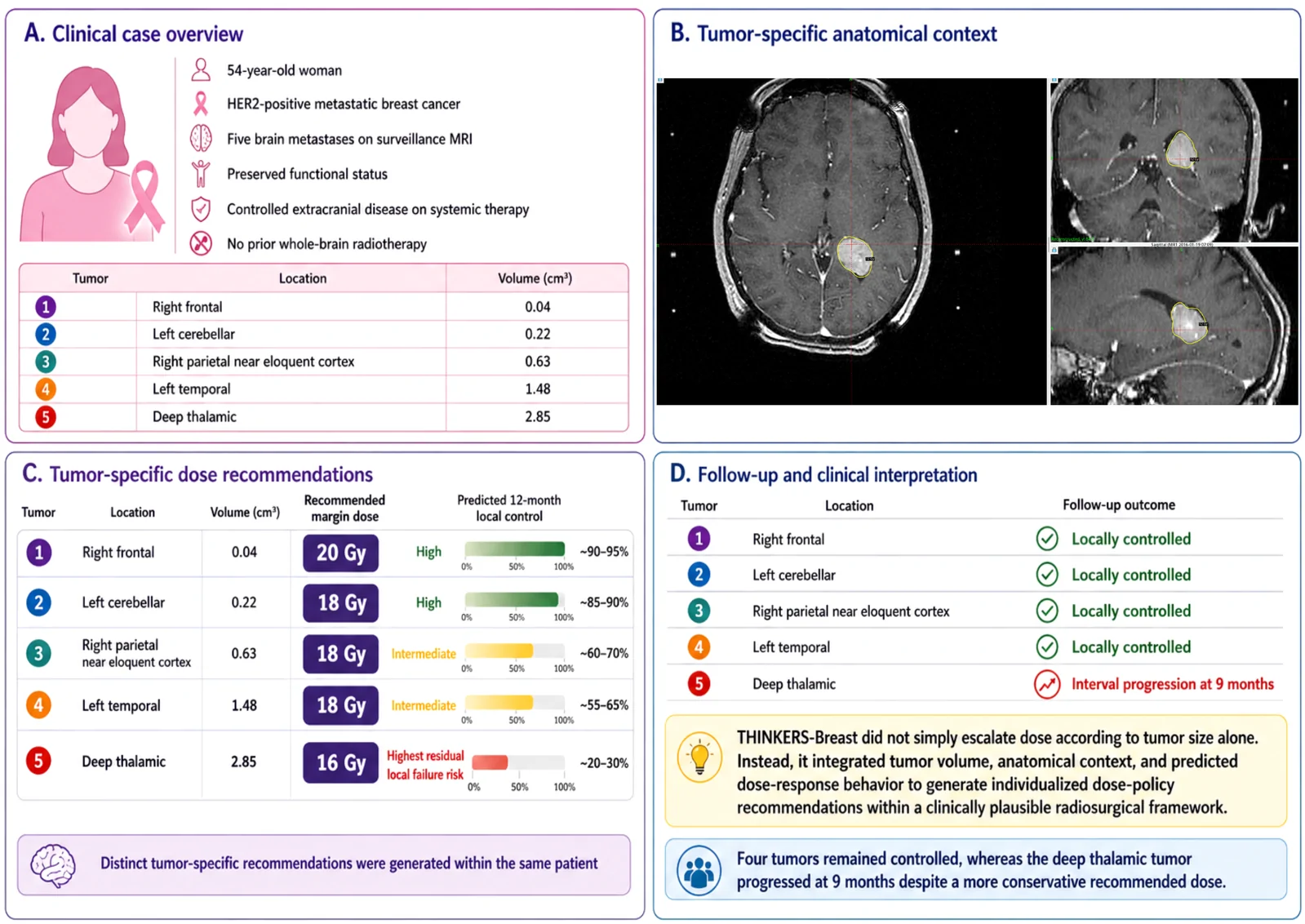
Illustrative case. Illustrative case of a 54-year-old woman with HER2-positive metastatic breast cancer treated with GKRS for five brain metastases. THINKERS-Breast generated tumor-specific margin dose recommendations of 20, 18, 18, 18, and 16 Gy based on tumor volume, anatomical context, and predicted dose-response behavior. Four tumors remained locally controlled, while the deep thalamic tumor progressed at 9 months.

THINKERS-Breast generated distinct tumor-specific dose recommendations rather than assigning a uniform prescription dose across all tumors. The framework recommended 20 Gy for the right frontal tumor, 18 Gy for the left cerebellar tumor, 18 Gy for the right parietal tumor near eloquent cortex, 18 Gy for the left temporal tumor, and 16 Gy for the deep thalamic tumor. The model predicted high 12-month local control for the two smallest tumors, intermediate control for the parietal and temporal tumors, and the highest residual local failure risk for the deep thalamic tumor despite a more conservative recommended dose. On follow-up, the frontal, cerebellar, parietal, and temporal tumors remained locally controlled, whereas the thalamic tumor demonstrated interval progression at 9 months. This example highlights that THINKERS-Breast does not simply escalate dose according to size alone, but integrates tumor volume, anatomical context, and dose-response behavior to generate individualized dose-policy recommendations within a clinically plausible radiosurgical framework (**Figure 4**).

## Discussion

In this study, we developed and internally validated THINKERS-Breast, a personalized AI framework for GKRS in breast cancer brain metastases that models time to local failure using a MoE architecture with discrete-time survival modeling. The final model demonstrated strong discrimination for 12-month local failure, favorable probabilistic performance, and clinically useful tumor-level outputs, including predicted local control over time and expected time to local failure. Taken together, these findings support the feasibility of using a tumor-specific AI framework not only to estimate local failure risk after GKRS, but also to provide comparative candidate dose evaluation for breast cancer brain metastases.

The central clinical problem addressed by this study is that prescription dose selection in radiosurgical practice remains largely guided by tumor size, location, proximity to critical structures, physician experience, and institutional convention (5–9). Although this approach is practical and clinically familiar, it does not formally estimate how a specific breast cancer brain metastasis may behave across different candidate margin doses (1–3). This distinction is important because two tumors from the same patient and same primary cancer may have very different volume, anatomical context, dose constraints, and predicted local control profiles. THINKERS-Breast was designed to address this gap by treating margin dose as a queryable input rather than as a fixed historical label. This allows the same tumor to be evaluated repeatedly across plausible candidate doses, making the framework more aligned with the actual decision-making process of GKRS planning.

A major strength of this framework is that it models time to local failure rather than reducing the outcome to a single binary label. Local control after SRS is inherently time dependent: some tumors remain controlled for prolonged periods, some fail early, and others fail later during follow-up. A discrete-time survival formulation allows the model to account for this temporal structure while incorporating censored observations more naturally than a purely binary classification approach. As a result, THINKERS-Breast generates clinically interpretable outputs beyond a yes-or-no prediction, including tumor-specific local control estimates over time, calibrated 12-month local failure risk, and expected time to local failure. These outputs are more relevant to post-SRS surveillance, patient counseling, and comparative treatment planning.

The use of a MoE architecture is conceptually appropriate for breast cancer brain metastases because this disease is biologically and clinically heterogeneous. Outcomes after GKRS may vary according to breast cancer subtype, systemic therapy exposure, intracranial disease burden, tumor volume, anatomical location, baseline functional status, and radiosurgical dose characteristics (1–7). A single predictive pathway may oversimplify these heterogeneous feature-dose-outcome relationships. In contrast, the MoE design allows multiple expert subnetworks to learn partially distinct patterns, while a gating mechanism adaptively weights their contributions for each tumor. This architecture may be particularly useful in breast cancer because clinically distinct biological states, including receptor subtype, systemic therapy exposure, intracranial disease burden, and lesion-specific radiosurgical characteristics, may not follow a single homogeneous dose-response relationship. By allowing individual experts to learn partially different relationships while the gating network assigns tumor-specific weights, the framework provides a flexible mechanism for representing both patient-level and lesion-level heterogeneity. From a clinical perspective, this structure parallels the multidisciplinary logic of radiosurgery, in which neurosurgical, radiation oncologic, and physics-based considerations are integrated before selecting a final treatment plan.

The choice of a neural network-based MoE architecture should be interpreted in relation to the clinical objective of the study. Simpler models such as logistic regression or random forest classifiers may be appropriate for single-endpoint prediction, but they provide less flexibility for modeling nonlinear dose-volume interactions, discrete-time local failure hazards, and repeated candidate dose evaluation (8–11). Similarly, traditional survival models may account for censoring but may not adequately capture heterogeneous and nonlinear treatment-response patterns across biologically distinct breast cancer brain metastases. In contrast, the MoE framework allowed THINKERS-Breast to generate tumor-specific expert weighting and dose-dependent local control estimates, making it better aligned with the personalized dose-evaluation goal of the study. This should not be interpreted as evidence that the MoE architecture is intrinsically superior to conventional statistical or machine-learning approaches, because these models were not trained as head-to-head comparators within the present cohort. Rather, the principal advantage of the architecture is its ability to integrate time-dependent prediction, heterogeneous feature-response relationships, and repeated candidate-dose querying within a unified framework.

Another important aspect of this study is that the final framework was restricted to variables available before or at the time of radiosurgery. Post-treatment variables and follow-up outcomes were excluded to prevent leakage and preserve clinical realism. This is essential for translational AI because models that use variables unavailable at the time of decision-making may appear accurate but fail as true clinical decision-support tools (1, 9).1 By relying on pre-treatment clinical, tumor, systemic therapy, imaging, and treatment-planning variables, THINKERS-Breast remains aligned with the information available during GKRS planning.

The performance metrics are encouraging. In grouped 5-fold cross-validation, the model achieved strong discrimination for 12-month local failure, with a raw mean AUC greater than 0.807 and a calibrated mean AUC of 0.864. In the grouped holdout test set, THINKERS-Breast achieved an AUC of 0.781 with a bootstrapped 95% confidence interval of 0.704–0.857, supporting meaningful performance in unseen patients and tumors. Probabilistic performance was also favorable, with raw and calibrated Brier scores below 0.14 and 0.16, respectively, and acceptable calibration intercepts ranging from -0.31 to +0.27. These findings suggest that the framework can meaningfully rank tumors according to local failure risk while also generating probability estimates suitable for clinical interpretation and comparative dose evaluation.

The feature-importance analysis provides additional insight into the relationships learned by the model. Margin dose and tumor volume were the strongest contributors to discrimination, a finding that is both clinically and biologically plausible. Tumor volume may reflect greater clonogenic tumor burden as well as increasing geometric and dosimetric complexity, whereas prescription margin dose directly determines the intensity of radiosurgical treatment delivered to the lesion. Their joint importance may therefore capture a clinically relevant dose-volume relationship in which larger tumors may be more difficult to achieve durable control while simultaneously being more likely to require conservative prescription dosing because of surrounding normal-tissue constraints. Importantly, these relationships should not be interpreted as purely causal, because historical dose selection itself is influenced by tumor size, location, prior treatment, and perceived toxicity risk.

Anatomical context may similarly influence predicted outcome through treatment-planning constraints rather than intrinsic radioresistance alone. Deep or eloquent-area lesions, including tumors involving or adjacent to the thalamus, internal capsule, brainstem, visual pathways, or other functionally critical structures, may narrow the therapeutic window because radiation-related injury in these regions can have substantial neurological consequences. This may limit the ability to escalate prescription dose in selected lesions. The predictive contribution of eloquence and anatomical location may therefore represent an interaction among tumor biology, volume, achievable dose, and the vulnerability of surrounding neural structures rather than an independent biological effect of location itself.

The dose-sweeping component is one of the most clinically relevant features of THINKERS-Breast. Many predictive models stop at risk stratification, but radiosurgical planning requires comparison among feasible treatment options. By repeatedly evaluating candidate margin doses for the same tumor, THINKERS-Breast reconstructs dose-dependent local control profiles and identifies individualized policy doses using a rule-based strategy. Importantly, the recommendation process does not simply favor the highest allowable dose or the lowest possible dose. Instead, it prioritizes longer predicted local failure-free time, followed by better calibrated 12-month local control, with lower dose used only as a final tie-breaker among near-equivalent candidates. This makes the framework better aligned with the practical logic of radiosurgery, where the goal is not automatic dose escalation or dose reduction, but selection of the most reasonable dose for a specific tumor.

Prior studies have evaluated prediction of local control, local failure, or treatment response after stereotactic radiotherapy for brain metastases. Kanakarajan et al. reported that a random forest model using clinical features alone achieved an AUC of 0.85 for local control prediction, while clinical-radiomics, clinical-deep learning, and combined clinical-radiomics-deep learning models achieved AUCs of 0.86, 0.82, and 0.88, respectively.19 Mouraviev et al. similarly showed that adding MRI radiomics to clinical features improved local control prediction after SRS, with an optimized clinical-radiomics model achieving a mean resampled AUC of 0.793.20 Other MRI radiomics models have reported AUCs of approximately 0.87 for local failure prediction after hypofractionated SRT, whereas a radiomic-based logistic model for local failure prediction achieved an independent holdout AUC of 0.78.21, 22 In comparison, THINKERS-Breast achieved a grouped holdout AUC of 0.781 and a C-index of 0.81. Although some image-intensive radiomics or deep learning models report higher discrimination, direct comparison based solely on AUC does not fully reflect differences in model purpose and functionality.

Beyond discrimination, several functional distinctions are relevant when comparing THINKERS-Breast with previous approaches. Most prior models were designed primarily to classify local control, local failure, or treatment response using clinical and/or imaging-derived features.19–23 They generally did not incorporate candidate prescription dose as a manipulable variable that could be repeatedly changed for lesion-level simulation. In addition, several prior approaches generated predictions at a single predefined endpoint or focused on post-treatment response classification, whereas THINKERS-Breast uses discrete-time survival modeling to generate interval-specific hazards and longitudinal local control estimates. The present framework was also developed specifically for breast cancer brain metastases rather than as a histology-agnostic model across heterogeneous primary cancers. Therefore, its contribution should not be interpreted as superior discrimination based on cross-study AUC comparison, but rather as the combination of histology-specific modeling, time-dependent outcome prediction, and individualized candidate dose evaluation within a single framework.

From a broader perspective, this study supports the development of histology-specific AI frameworks for computational radiosurgery. Breast cancer brain metastases are not a uniform clinical entity, and treatment response may be shaped by tumor biology, systemic therapy context, and tumor-level radiosurgical factors. A breast-specific model may therefore provide more relevant decision support than a broad, one-size-fits-all brain metastasis model. The significance of THINKERS-Breast lies not only in its predictive performance, but also in its structure as a potentially actionable framework that connects patient and tumor characteristics, candidate margin dose, predicted local control, and expected time to local failure.

The broader implication is that AI in radiosurgery may ultimately be most useful when it formalizes treatment comparisons that clinicians already make in practice rather than replacing clinical judgment. THINKERS-Breast was designed with that objective in mind. By integrating pre-treatment variables with candidate margin dose and generating tumor-specific local control estimates, the framework provides a structured approach for individualized dose-policy auditing. However, the present findings represent internal validation from a single-center retrospective cohort and should therefore be considered hypothesis-generating. Multicenter external validation and prospective evaluation will be required to determine whether these predictions remain reliable across institutions, treatment practices, and patient populations and whether they can ultimately provide meaningful clinical decision support.

### Limitations

This study has several limitations. First, it was a retrospective single-center analysis and may reflect institutional treatment patterns, follow-up practices, imaging interpretation, and dose-selection preferences that limit generalizability. Second, although grouped patient-level validation was used to reduce tumor-level leakage, the framework remains internally validated only and requires external multicenter validation before clinical implementation. Third, prescription dose was selected by treating physicians rather than assigned randomly, so the dose-sweeping module should be interpreted as a decision-support and dose-policy auditing tool rather than a causal estimator of the optimal dose. Fourth, local failure assessment and follow-up intervals were subject to retrospective heterogeneity. Fifth, conventional approaches such as Cox regression, random forest, and gradient boosting were not trained as head-to-head comparators within the same cohort. Therefore, the present study cannot establish superior predictive performance of the MoE architecture over these methods, and comparisons with previously published models should be interpreted as cross-study contextualization. Finally, systemic therapy exposure, breast cancer subtype, molecular status, and treatment timing may have effects that are more complex than can be completely represented in a structured dataset.

## Conclusions

THINKERS-Breast is an internally validated mixture-of-experts discrete-time survival framework for tumor-specific local failure prediction and comparative dose evaluation after GKRS for breast cancer brain metastases. The model demonstrated meaningful discrimination, favorable probabilistic performance, and clinically interpretable outputs, including time-dependent local control estimates, expected time to local failure, and candidate dose-specific predictions. These findings support the feasibility of histology-specific, lesion-level AI modeling in radiosurgery and lay a foundation for subsequent multicenter external validation and prospective clinical translation studies. Clinical implementation should not be considered until the framework has been independently validated across institutions and patient populations.

## Data Availability

Requests to access these datasets should be directed to reyesbarretojs@upmc.edu.
