# Supplementary material for: Therapeutic hybrid intelligence with neural and knowledge-based expert reasoning for SRS: an AI model for breast cancer brain metastases
Source: Front Oncol. 2026 Sep 11;16:1884891. doi: 10.3389/fonc.2026.1884891 (PMC13612181; doi:10.3389/fonc.2026.1884891)
Supplement: Supplementary file 1 [file Table1.docx]

Supplementary Table 1. Input variable dictionary and preprocessing strategy for THINKERS-Breast.

| **Variable** | **Definition** | **Level** | **Data type** | **Possible values / range** | **Preprocessing / missing data handling** |
| --- | --- | --- | --- | --- | --- |
| **Patient-level clinical variables** |  |  |  |  |  |
| Age at treatment | Patient age at GKRS | Patient | Continuous | Years | Median imputation; standardization |
| Sex | Biological sex | Patient | Categorical | Female, male | Missing values encoded as separate category; one-hot encoding |
| Race | Recorded race | Patient | Categorical | White, African American, Asian, other/missing | Missing values encoded as separate category; one-hot encoding |
| Pre-treatment KPS | Karnofsky Performance Status before GKRS | Patient | Continuous | 10–100 | Median imputation; standardization |
| **Breast cancer biology and systemic therapy variables** |  |  |  |  |  |
| Breast cancer subtype / specific tumor type | Recorded breast cancer subtype or histologic category | Patient/tumor | Categorical | Adenocarcinoma, triple negative, triple positive, ductal, other | Missing values encoded as separate category; one-hot encoding |
| Systemic therapy status | Whether systemic therapy was recorded at treatment time | Patient | Categorical | Yes, no | Missing values encoded as separate category; one-hot encoding |
| Systemic therapy specification | Specific systemic therapy category or agent when available | Patient | Categorical | Agent/class as documented; missing/unknown | Missing values encoded as separate category; one-hot encoding with infrequent levels grouped |
| **Additional clinical and prior treatment variables** |  |  |  |  |  |
| Extracranial metastatic disease | Presence of metastatic disease outside the brain at or before GKRS | Patient | Categorical | Yes, no, unknown | Missing values encoded as separate category; one-hot encoding |
| Prior WBRT | History of whole-brain radiotherapy before GKRS | Patient | Categorical | Yes, no, unknown | Missing values encoded as separate category; one-hot encoding |
| Prior SRS | History of stereotactic radiosurgery before the indexed GKRS treatment | Patient/tumor | Categorical | Yes, no, unknown | Missing values encoded as separate category; one-hot encoding |
| Prior neurosurgery | History of prior neurosurgical resection or procedure for brain metastasis before GKRS | Patient | Categorical | Yes, no, unknown | Missing values encoded as separate category; one-hot encoding |
| Prior targeted therapy | History of targeted systemic therapy for breast cancer before or at GKRS | Patient | Categorical | Yes, no, unknown | Missing values encoded as separate category; one-hot encoding |
| Breast cancer recurrence | Documented recurrent breast cancer before or at GKRS | Patient | Categorical | Yes, no, unknown | Missing values encoded as separate category; one-hot encoding |
| Recurrent brain metastasis | Whether the treated tumor or intracranial disease represented recurrent brain metastasis | Patient/tumor | Categorical | Yes, no, unknown | Missing values encoded as separate category; one-hot encoding |
| Symptomatic status at GKRS | Presence of neurological symptoms at the time of GKRS | Patient | Categorical | Symptomatic, asymptomatic, unknown | Missing values encoded as separate category; one-hot encoding |
| Prior treatment failure | History of prior intracranial treatment failure before the indexed GKRS treatment | Patient/tumor | Categorical | Yes, no, unknown | Missing values encoded as separate category; one-hot encoding |
| Retreatment history | Whether GKRS was performed in the setting of retreatment | Patient/tumor | Categorical | Yes, no, unknown | Missing values encoded as separate category; one-hot encoding |
| **Tumor-level variables** |  |  |  |  |  |
| Number of metastases | Intracranial metastasis burden at GKRS | Patient | Categorical or ordinal | Single, multiple | Missing values encoded as separate category; one-hot encoding |
| Metastasis location | Broad intracranial compartment | Tumor | Categorical | Supratentorial, infratentorial | Missing values encoded as separate category; one-hot encoding |
| Lobe / structure | Anatomical tumor location | Tumor | Categorical | Cerebellum, frontal, parietal, temporal, occipital, other | Missing values encoded as separate category; one-hot encoding |
| Side | Tumor laterality | Tumor | Categorical | Left, right | Missing values encoded as separate category; one-hot encoding |
| Eloquence | Whether tumor involved or was adjacent to eloquent/critical anatomy | Tumor | Categorical | Yes, no | Missing values encoded as separate category; one-hot encoding |
| Tumor volume | Treated tumor volume | Tumor | Continuous | 0.002–50.159 cm³ | Median imputation; standardization |
| **GKRS treatment and planning variables** |  |  |  |  |  |
| Immobilization | GKRS immobilization approach | Treatment | Categorical | Frame, mask, CT-based setup/other | Missing values encoded as separate category; one-hot encoding |
| Imaging technique | Imaging modality used for planning | Treatment | Categorical | MRI, CT | Missing values encoded as separate category; one-hot encoding |
| Margin dose | Prescription margin dose | Treatment | Continuous / queryable | 8–27 Gy in historical cohort | Median imputation; standardization; used as queryable input for dose sweeping |
| Isodose line | Prescription isodose line | Treatment | Continuous | 40–90% | Median imputation; standardization |
| Dose to target volume | Dose prescribed to target volume | Treatment | Continuous | 8–27 Gy | Median imputation; standardization |
| Minimum dose | Minimum dose to target | Treatment | Continuous | 0.5–147 Gy | Median imputation; standardization |
| Maximum dose | Maximum dose to target | Treatment | Continuous | 0.6–520.3 Gy | Median imputation; standardization |
| Mean dose | Mean dose to target | Treatment | Continuous | 0.6–241.9 Gy | Median imputation; standardization |
| Number of collimators | Number of collimators or shots used during planning | Treatment | Continuous / count | 1–124 | Median imputation; standardization |
| Treatment duration | Total treatment time | Treatment | Continuous | 1–191 minutes | Median imputation; standardization |
| **Outcome variables** |  |  |  |  |  |
| Local failure time | Time from GKRS to documented local failure or censoring | Outcome | Continuous | Follow-up dependent | Not used as model input |
| Local failure status | Whether treated tumor developed local failure | Outcome | Binary | Event, censored/controlled | Not used as model input |
