# Supplementary material for: Therapeutic hybrid intelligence with neural and knowledge-based expert reasoning for SRS: an AI model for breast cancer brain metastases
Source: Front Oncol. 2026 Sep 11;16:1884891. doi: 10.3389/fonc.2026.1884891 (PMC13612181; doi:10.3389/fonc.2026.1884891)
Supplement: Supplementary file 2 [file Table2.docx]

**Supplementary Table 2**. Ranked grouped feature-contribution analysis for THINKERS-Breast.

| **Rank** | **Variable / variable group** | **Variable category** | **Baseline AUC** | **AUC after permutation** | **ΔAUC** | **Relative importance (%)** |
| --- | --- | --- | --- | --- | --- | --- |
| 1 | Margin dose | Radiosurgical dose | 0.781 | 0.726 | 0.055 | 100 |
| 2 | Tumor volume | Tumor burden / size | 0.781 | 0.732 | 0.049 | 89 |
| 3 | Dose-volume interaction features | Derived radiosurgical features | 0.781 | 0.738 | 0.043 | 78 |
| 4 | Mean dose / dose to target volume | Radiosurgical dose distribution | 0.781 | 0.749 | 0.032 | 58 |
| 5 | Maximum dose and dose heterogeneity | Radiosurgical dose distribution | 0.781 | 0.752 | 0.029 | 53 |
| 6 | Minimum dose and minimum-dose-to-margin ratio | Radiosurgical dose distribution | 0.781 | 0.755 | 0.026 | 47 |
| 7 | Number of metastases | Intracranial tumor burden | 0.781 | 0.759 | 0.022 | 40 |
| 8 | Lobe / anatomical structure | Anatomical context | 0.781 | 0.762 | 0.019 | 35 |
| 9 | Eloquence | Anatomical / treatment constraint | 0.781 | 0.764 | 0.017 | 31 |
| 10 | Breast cancer subtype / specific tumor type | Tumor biology | 0.781 | 0.766 | 0.015 | 27 |
| 11 | Systemic therapy status | Systemic disease / treatment context | 0.781 | 0.768 | 0.013 | 24 |
| 12 | Systemic therapy specification | Systemic disease / treatment context | 0.781 | 0.769 | 0.012 | 22 |
| 13 | Isodose line | Radiosurgical planning | 0.781 | 0.771 | 0.010 | 18 |
| 14 | Treatment duration | Treatment planning complexity | 0.781 | 0.773 | 0.008 | 15 |
| 15 | Number of collimators | Treatment planning complexity | 0.781 | 0.774 | 0.007 | 13 |
| 16 | Pre-treatment KPS | Clinical status | 0.781 | 0.775 | 0.006 | 11 |
| 17 | Age at treatment | Clinical status | 0.781 | 0.776 | 0.005 | 9 |
| 18 | Metastasis location: supratentorial vs infratentorial | Anatomical context | 0.781 | 0.777 | 0.004 | 7 |
| 19 | Side | Anatomical context | 0.781 | 0.778 | 0.003 | 5 |
| 20 | Immobilization approach | Treatment setup | 0.781 | 0.779 | 0.002 | 4 |
| 21 | Imaging technique | Treatment setup | 0.781 | 0.779 | 0.002 | 4 |
| 22 | Sex | Clinical variable | 0.781 | 0.780 | 0.001 | 2 |
| 23 | Race | Clinical variable | 0.781 | 0.780 | 0.001 | 2 |
