# Supplementary material for: Therapeutic hybrid intelligence with neural and knowledge-based expert reasoning for SRS: an AI model for breast cancer brain metastases
Source: Front Oncol. 2026 Sep 11;16:1884891. doi: 10.3389/fonc.2026.1884891 (PMC13612181; doi:10.3389/fonc.2026.1884891)
Supplement: Supplementary file 3 [file Table3.docx]

**Supplementary Table 3.** Derived dose-volume variables used in THINKERS-Breast.

| **Derived variable** | **Original variables used** | **Definition / formula** | **Rationale** | **Contribution to model** |
| --- | --- | --- | --- | --- |
| Log tumor volume | Tumor volume | log(1 + tumor volume in cm³) | Tumor volume was highly right-skewed. Log transformation reduces the influence of extreme values while preserving relative differences among small tumors. | Very high |
| Tumor volume category | Tumor volume | Tumors categorized into volume-based groups according to the distribution of treated tumor volumes | Allows the model to capture nonlinear size effects that may not be fully represented by continuous volume alone. | High |
| Margin dose × log tumor volume | Margin dose; log tumor volume | Margin dose × log(1 + tumor volume) | Captures dose-volume interaction, recognizing that the effect of a given margin dose may differ between small and large tumors. | Very high |
| Dose to target volume × log tumor volume | Dose to target volume; log tumor volume | Dose to target volume × log(1 + tumor volume) | Represents interaction between delivered target dose and tumor size. | High |
| Margin dose-to-volume ratio | Margin dose; tumor volume | Margin dose / (1 + tumor volume) | Represents relative prescription dose intensity normalized to tumor size. | High |
| Dose to target volume-to-volume ratio | Dose to target volume; tumor volume | Dose to target volume / (1 + tumor volume) | Captures delivered dose intensity relative to tumor burden. | Moderate to high |
| Minimum dose-to-margin dose ratio | Minimum dose; margin dose | Minimum dose / margin dose | Reflects lower-bound target coverage relative to the prescribed margin dose. | High |
| Maximum dose-to-margin dose ratio | Maximum dose; margin dose | Maximum dose / margin dose | Represents the upper range of delivered dose relative to prescription dose and may reflect dose heterogeneity. | High |
| Mean dose-to-margin dose ratio | Mean dose; margin dose | Mean dose / margin dose | Captures average delivered dose intensity relative to prescription dose. | High |
| Dose heterogeneity range | Maximum dose; minimum dose | Maximum dose − minimum dose | Represents spread of dose within the treated tumor and overall heterogeneity of dose delivery. | Moderate to high |
| Mean-to-minimum dose ratio | Mean dose; minimum dose | Mean dose / minimum dose | Provides a relative measure of dose distribution and potential undercoverage. | Moderate |
| Maximum-to-mean dose ratio | Maximum dose; mean dose | Maximum dose / mean dose | Captures hotspot intensity relative to average target dose. | Moderate |
